# Study on Detection Method of Sulfamethazine Residues in Duck Blood Based on Surface-Enhanced Raman Spectroscopy

**DOI:** 10.3390/bios15050286

**Published:** 2025-05-01

**Authors:** Junshi Huang, Runhua Zhou, Jinlong Lin, Qi Chen, Ping Liu, Shuanggen Huang, Jinhui Zhao

**Affiliations:** 1 Key Laboratory of Modern Agricultural Equipment of Jiangxi, Jiangxi Agricultural University, Nanchang 330045, China; huangjunshi@jxau.edu.cn (J.H.); z136979357777@sina.com (R.Z.); rincy@jxau.edu.cn (J.L.); kaikai567726@sina.com (Q.C.); 2 College of Animal Science and Technology, Jiangxi Agricultural University, Nanchang 330045, China; pingliu@jxau.edu.cn

**Keywords:** duck blood, surface-enhanced Raman spectroscopy, sulfamethazine, density functional theory

## Abstract

Sulfadimethazine (SM2) is widely used in livestock and poultry farming, but its improper use can pose a serious threat to human health. Therefore, the detection of SM2 residues in livestock and poultry products, including duck blood, is of great significance for food safety. A rapid detection method for SM2 residues in duck blood based on surface-enhanced Raman spectroscopy (SERS) was proposed in this paper. Density functional theory (DFT) was employed to optimize the molecular structure of SM2 and perform theoretical Raman vibrational analysis, thereby identifying its characteristic peaks. The enhancement effects of four different substrates were compared. The sample pretreatment method and detection conditions were optimized through single-factor experiments, including the types and amounts of electrolyte aggregators, the amount of gold nanocolloids, and the adsorption time. Under optimal conditions, the SERS spectral data of the samples were preprocessed, and features were extracted to establish an optimal quantitative prediction model. The experimental results found that the adaptive iteratively reweighted penalized least-squares method (air-PLS) was the best preprocessing method, and the competitive adaptive reweighted sampling–multiple linear regression (CARS-MLR) model demonstrated the best prediction performance, with a coefficient of determination for the prediction set (R_p_^2^) of 0.9817, a root mean square error of calibration (RMSEC) of 1.5539 mg/L, a relative prediction deviation (RPD) of 7.1953, and limits of quantification of 0.75 mg/L. The research demonstrated that the combination of SERS technology and chemometric methods was feasible and effective for the detection of SM2 residues in duck blood.

## 1. Introduction

Sulfamethazine (SM2) is a synthetic antibacterial agent that belongs to the class of broad-spectrum sulfonamide antibiotics. It is widely applied in veterinary medical practice and is frequently used as an antibiotic growth promoter in livestock and poultry [1,2]. However, the excessive and unreasonable use of SM2 may give rise to a range of public health issues and potentially pose a threat to human health [3,4,5,6,7]. The conventional detection methods for SM2 currently include microbiological methods, high-performance liquid chromatography, and liquid chromatography–tandem mass spectrometry, among others. These methods are characterized by high accuracy and sensitivity, but the pretreatment is complex, the cost is high, and the detection speed is slow, making them unsuitable for on-site rapid detection and screening [8,9,10]. As a convenient, efficient, and practical modern spectral detection and analysis method, surface-enhanced Raman spectroscopy (SERS) has been widely used in chemical detection, biomedicine, and environmental analysis [11,12]. Recently, some scholars at home and abroad have also carried out extensive research on the SERS detection of veterinary drug residues in food. Based on SERS technology, Xu et al. prepared silver nanocolloids to detect penicillin G sodium, amoxicillin, and chloramphenicol residues in poultry meat, and good detection results were obtained [13]. Wang et al. classified doxycycline hydrochloride, tylosin, ofloxacin, and norfloxacin residues in chicken and duck meat based on SERS coupled with chemometric models [14]. Natalia et al. used cyclodextrins (CDs) to improve the effect of surface-enhanced Raman spectroscopy for the determination of fluoroquinolone antibiotics in body fluids. By studying surface-enhanced Raman spectroscopy [15], Lee et al. established a rapid, non-destructive, and reliable analysis method for chlortetracycline and oxytetracycline in animal feed. The results showed that SERS could be used as an alternative or supplementary technique for the rapid determination of tetracycline in feed [16]. In this study, duck blood was used as a carrier, and SM2 was used as the research object. The purpose of this study was to establish a rapid detection method for SM2 residues by combining SERS technology and chemometrics methods and to provide new ideas and technical means for veterinary drug residue monitoring.

## 2. Materials and Methods

### 2.1. Materials and Reagents

Duck blood samples were collected from five different vendors at the agricultural market of Jiangxi Agricultural University, totaling 20 fresh specimens. Through breed traceability verification, all samples were confirmed to originate from Cherry Valley ducks raised in standardized commercial farms. This breed is internationally recognized as a mainstream meat-type duck, characterized by short growth cycles and low feed conversion ratios. Immediately after collection, all samples were stored at 4 °C to preserve their biological activity. A 50 mL duck blood sample was sent to a professional testing institution to test, and no SM2 in duck blood was detected using the national food safety standard (maximum residue limit of veterinary drug in food, GB31650) and liquid chromatography–mass spectrometry (Agilent Technology Co., Ltd., Santa Clara, CA, USA) [17].

Thylenediamine tetraacetic acid-dipotassium (EDTA-2K) with a purity of 10× was purchased from Yuanye Biological Technology Co., Ltd. (Shanghai, China). SM2 (≥98%) and formic acid (≥88%) were purchased from Aladdin Biochemical Technology Co., Ltd. (Shanghai, China). Acetonitrile (analytical grade), sodium chloride (NaCl, analytical grade), calcium chloride (CaCl_2_, analytical grade), magnesium sulfate (MgSO_4_, analytical grade), potassium bromide (KBr, analytical grade), trisodium citrate dihydrate (Na_3_C_6_H5O_7_·2H_2_O, analytical grade), and silver nitrate (AgNO_3_, analytical grade) were obtained from Xilong Scientific Co., Ltd. (Shantou, China). Tetrachloroauric acid trihydrate (HAuCl_4_·3H_2_O, ACS level) was purchased from Sigma-Aldrich Trading Co., Ltd. (Shanghai, China).

### 2.2. Preparation of Four Enhancement Substrates

According to the Frens method, an SERS-enhanced substrate was synthesized using tetrachloroauric acid trihydrate (HAuCl_4_·3H_2_O) and sodium citrate solution. First, 100 mL of 1 mM HAuCl_4_ solution was added to a round-bottom flask and heated to boiling. Then, immediately after, 3.7 mL of 1% trisodium citrate solution was added. The mixture was continuously heated and stirred for 30 min using a ZNCL-T intelligent constant temperature agitator (Zhengzhou Yarong Instrument Co., Ltd., Zhengzhou, China). The resulting reddish-brown suspensions of AuNPs were then cooled to room temperature and stored at 4 °C [18]. The enhanced substrate prepared by this method is called 1 mM gold nanocolloids in this paper.

To prepare 1% gold nanocolloids, 100 mL of HAuCl_4_ solution with a concentration of 0.01% was heated to boil, and 1 mL trisodium citrate solution with a concentration of 1% was immediately added for heating by stirring using a magnetic stirrer for 15 min. The resulting colloid was cooled to room temperature for later use [19]. The enhanced substrate prepared by this method is called 1% gold nanocolloids in this paper.

To prepare 14th-generation gold nanocolloids, 150 mL of trisodium citrate solution with a concentration of 2.2 mM was placed into a three-necked round-bottom flask to heat to boiling, and then 1 mL of 25 mM HAuCl_4_ solution was added. After 10 min, the solution changed from yellow to light pink. Subsequently, the temperature in the flask was controlled at 90 °C, and 1 mL of 60 mM sodium citrate solution was immediately added. After 2 min, 1 mL of 25 mM HAuCl_4_ solution was added. After 30 min, 2 mL of the solution was removed from the flask. After repeating the above process 13 times while controlling the temperature at 90 °C, the resulting colloid was obtained and named 14th-generation gold nanocolloids in this paper. The colloid was then cooled to room temperature for later use [20].

To prepare silver nancolloids, 100 mL of AgNO_3_ solution with a concentration of 0.001 M was placed in a three-necked round-bottom flask. After stirring and heating to boil, 2 mL of trisodium citrate solution with a concentration of 1% was immediately added. The solution changed from colorless to pale yellow. After 1 h, the solution gradually became dark gray, and the obtained colloid was cooled to room temperature and stored in the dark for later use [21]. The enhanced substrate prepared by this method is called silver nanocolloids in this paper.

### 2.3. Duck Blood Sample Preparation

The obtained fresh duck blood was placed in a 100 mL centrifuge tube (with anticoagulant added beforehand; the volume ratio of anticoagulant to duck blood was 1:9) and stored at low temperature for later use.

Then, 1 mL of formic acid solution was placed in a 1000 mL brown volumetric flask, which was then diluted to 1000 mL with acetonitrile solution. The mixture was mixed evenly to obtain a 0.1% formic acid acetonitrile solution for standby.

A total of 8 mL of blood was collected in a 50 mL centrifuge tube, and 2 mL of SM2 standard solution at different concentrations was added. The mixture was then thoroughly mixed, vortexed using a VORTEX-6 whirlpool mixer (Haimen Kylin-Bell Lab Instruments Co., Ltd., Nantong, China) for 2 min, and sonicated with a JK-50B ultrasonic cleaner (Hefei Jinnick Machinery Manufacturing Co., Ltd., Hefei, China) for 5 min to prepare blood samples with varying concentrations.

We took 10 mL of 0.1% formic acid in acetonitrile solution and added it separately to blood samples of different concentrations. After mixing thoroughly, the mixtures were vortexed for 30 s and then sonicated for 5 min. Then, the mixtures were placed into a JW-1024 centrifuge (Anhui Jiawen Instrument Equipment Co., Ltd., Hefei, China) and centrifuged at 4500 rpm for 10 min. The supernatants were collected for subsequent detection.

### 2.4. Optimization of Sample Pretreatment

During the sample pretreatment process, the volume ratio of the blood samples to 0.1% formic acid acetonitrile solution may influence the extraction efficiency of antibiotics. Therefore, SERS spectra of duck blood samples containing 5 mg/L of SM2 were compared at five different ratios of duck blood to extractant (i.e., 2:3, 4:5, 1:1, 4:3, 2:1) in order to determine the optimal ratio.

### 2.5. Optimization Scheme of SERS Detection Conditions

By comparing the SERS spectra of duck blood samples containing 5 mg/L of SM2, blank duck blood samples, 0.1% formic acid acetonitrile solution, and the enhanced substrate, SERS characteristic peaks at 588 cm^−1^, 1005 cm^−1^, and 1593 cm^−1^ were obtained for identifying SM2 residues in duck blood.

#### 2.5.1. Optimization of Electrolyte Types

To investigate the effect of different electrolytes on the SERS spectrum of duck blood samples containing 5 mg/L SM2, 550 μL of 1 mM gold nanocolloids and 20 μL of duck blood were added to a quartz flask. Subsequently, 75 μL of 0.1 mol/L CaCl_2_ solution, 0.1 mol/L MgSO_4_ solution, 0.1 mol/L NaCl solution, 0.1 mol/L KBr solution, and ultrapure water were added separately. After thorough mixing, SERS detection was performed on five replicate samples for each electrolyte-treated mixture following a 1 min adsorption period. The average spectral value was calculated to represent the SERS spectrum of each sample. The optimal electrolyte was determined by comparing the average intensities of characteristic peaks at 588 cm^−1^, 1005 cm^−1^, and 1593 cm^−1^ under the five different electrolyte conditions.

#### 2.5.2. Optimization of Electrolyte Addition Amount

To investigate the effect of varying electrolyte additions on the SERS spectrum of duck blood samples containing 5 mg/L SM2, 550 μL of 1 mM gold nanocolloids, and 20 μL of duck blood were combined in a quartz flask. Subsequently, 25 μL, 50 μL, 75 μL, 100 μL, and 125 μL of 0.1 mol/L CaCl_2_ solution were added, respectively. After thorough mixing, SERS detection was conducted on five identical samples for each electrolyte addition level, following a 1 min adsorption period. The average spectral value was calculated to represent the SERS spectrum for each sample set. By comparing the average intensities of the characteristic peaks at 588 cm^−1^, 1005 cm^−1^, and 1593 cm^−1^ across the five different electrolyte addition levels, the optimal amount of electrolyte addition was determined. Similarly, by comparing the average intensities of these characteristic peaks under five different electrolyte types, the most effective electrolyte type was identified based on the peak intensities.

#### 2.5.3. Optimization of Addition Amount of 1 mM Gold Nanocolloids

To investigate the effect of varying amounts of 1 mM gold nanocolloid solution on the SERS spectrum of duck blood samples containing 5 mg/L SM2, quartz flasks were prepared with 450 μL, 500 μL, 550 μL, 600 μL, and 650 μL of 1 mM gold nanocolloid solution, along with 20 μL of duck blood samples and 75 μL of 0.1 mol/L CaCl_2_ solution, respectively. The mixtures were then thoroughly combined. Following a 1 min adsorption period, SERS spectra were obtained from five identical samples for each gold nanocolloid solution amount. The average spectrum was considered the representative SERS spectrum for each sample. By comparing the average intensities of the characteristic peaks at 588 cm^−1^, 1005 cm^−1^, and 1593 cm^−1^ across the five different amounts of 1 mM gold nanocolloid solution, the optimal addition amount was determined based on the peak intensities.

#### 2.5.4. Optimization of Adsorption Time

To investigate the effect of varying adsorption times on the SERS spectrum of duck blood samples containing 5 mg/L SM2, a quartz flask was prepared with 550 μL of 1 mM gold nanocolloids, 20 μL of duck blood, and 75 μL of 0.1 mol/L CaCl_2_ solution, and the mixture was thoroughly homogenized. SERS spectra were collected from five parallel samples following adsorption periods of 0, 1, 2, 3, and 4 min, respectively. By comparing the mean intensities of the characteristic peaks at 588 cm^−1^, 1005 cm^−1^, and 1593 cm^−1^ across the five conditions, the optimal adsorption time for the application of 1 mM gold nanocolloids was established.

### 2.6. Quantitative Detection Scheme of SM2 in Duck Blood

A total of 120 duck blood samples containing SM2 were prepared within the concentration range of 0.75 mg/L to 45 mg/L, with each sample being tested once. For SERS spectrum measurement, a quartz vial was filled with 550 μL of 1 mM gold nanocolloids, 20 μL of the duck blood sample, and 75 μL of CaCl_2_ solution. After thorough mixing and a 1 min adsorption period, the mixture was placed on the sample detection platform of a portable Raman spectrometer (Ocean Optics Technology Co., Ltd., Orlando, FL, USA) for analysis. The SERS spectrum spanning from 400 to 1800 cm^−1^ was selected for data interpretation. Out of the 120 samples, 90 were randomly selected to constitute the training set for developing the prediction model, while the remaining 30 samples were used as the test set for quantitative prediction.

### 2.7. Density Functional Theory Calculations and Spectral Acquisition of SM2 Standard

The molecular structure of SM2 was optimized using Gaussian software (Gaussian 09, GaussView 05) through density functional theory calculations with the B3LYP hybrid functional and 6-311+G(d,p) basis set, followed by theoretical Raman spectral calculations. The Raman spectra of the standard solid sample were acquired using a DXR™ micro-Raman spectrometer (Thermo Fisher Scientific Co., Ltd., Waltham, MA, USA). An appropriate amount of antibiotic standard powder was placed on a glass slide, gently pressed, and positioned on the spectrometer stage for measurement under a 10× objective lens. The instrumental parameters were set as follows: laser power at 8 mW, acquisition time of 10 s, exposure time of 5 s, with 16 background exposures and 10 sample exposures.

### 2.8. SERS Spectral Acquisition of SM2 in Duck Blood

To measure the SERS spectrum, a predetermined volume of the enhanced substrate, 20 μL of the sample under test, and a specific volume of electrolyte solution were sequentially introduced into a quartz bottle. Following mixing and adsorption for a designated period, the mixture was positioned on the sample detection platform of a portable Raman spectrometer to acquire both qualitative and quantitative SERS spectra of duck blood samples. During sample collection, the integration time was set to 10 s, the laser power was adjusted to 850 mW, data were averaged over 2 scans, and the smoothness parameter was set to 1. For the purpose of data analysis, SERS spectra within the range of 400 to 1800 cm^−1^ were selected.

### 2.9. Data Processing Method

In this paper, the adaptive iteratively reweighted penalized least-squares method (air-PLS) implemented in MATLAB R2014b was utilized to correct the influence of background and drift on the original spectral data. Subsequently, the first and second derivatives were applied to further mitigate the effects of background and drift. Combined with the Savitzky–Golay (SG) filter, the spectrum was smoothed by removing baseline offset and resolving resolution overlap. Furthermore, the use of multiplicative scatter correction (MSC) and standard normalized variate (SNV) helped enhance the correlation between spectra by diminishing the impact of errors. By incorporating normalization, the spectral variability arising from light scattering was effectively eliminated [22,23,24].

To reduce the number of input variables for the model, two feature extraction methods, namely competitive adaptive reweighted sampling (CARS) and principal component analysis (PCA), were compared and analyzed. Utilizing MATLAB software, multiple iterations of adaptive weighted sampling were conducted with CARS, allowing for the selection of characteristic bands from a large set of preprocessed spectral bands to serve as inputs for the quantitative prediction model [25,26]. PCA provides a powerful tool for extracting unsupervised features from large datasets. As it extracts relevant information and reduces the dimensionality of the dataset, it is also frequently applied to the analysis of spectral data [27,28,29]. In Unscrambler X software, the PCA method was applied to dimensionally reduce the preprocessed spectral data, employing a variable reduction approach on the obtained dataset. The original data matrix was transformed into a new, lower-dimensional space. The new variables, constituting the model, were termed principal components, which captured the majority of the variability present in the original dataset. These principal components were then utilized as inputs for establishing the quantitative prediction model.

Following the preprocessing of the original spectral data and the mitigation of unfavorable factors, the dataset underwent further reduction through feature extraction. Ultimately, the most optimal quantitative prediction model was developed. In this study, the prediction performances of three quantitative models—partial least squares regression (PLSR), multiple linear regression (MLR), and support vector machine (SVM)—were compared. These models were assessed using Unscrambler X software for their capability to predict SM2 residues in duck blood. By examining various evaluation metrics, including the coefficient of determination for the calibration set (R_c_^2^), the coefficient of determination for the prediction set (R_p_^2^), the root mean square error of the calibration set (RMSEC), the root mean square error of the prediction set (RMSEP), and the relative prediction deviation (RPD), the best model for detecting SM2 residues in duck blood was determined.

## 3. Results and Discussion

### 3.1. Analysis of Raman Characteristic Peak Spectrum and Peak Position Attribution of SM2

**Figure 1:**
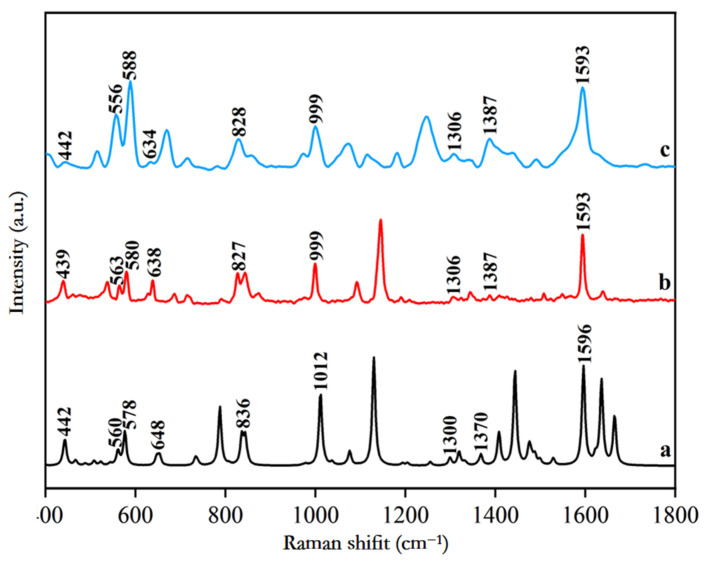
Raman spectrum comparison of SM2. (**a**) Theoretical Raman spectroscopy; (**b**) Raman spectrum of SM2 standard product; (**c**) SERS spectrum of standard aqueous solution with 5 mg/L.

The molecular structure of SM2 was optimized and theoretically calculated using Gaussian software, with convergent computational results. The theoretically calculated Raman spectrum obtained after optimization is presented in Figure 1a. Simultaneously, the experimental Raman spectrum of the solid SM2 standard was acquired using a DXR™ micro-Raman spectrometer (Thermo Fisher Scientific Co., Ltd., Waltham, MA, USA), as shown in Figure 1b. For surface-enhanced Raman scattering experiments, 500 μL of 1 mM gold nanocolloids enhancement substrate was mixed with 20 μL of 5 mg/L SM2 standard solution in a quartz vial. The mixture was thoroughly vortexed for 1 min to ensure uniform adsorption before being placed on the sample detection platform of a portable Raman spectrometer for measurement. The resulting SERS spectrum is displayed in Figure 1c.

From Figure 1, it is evident that the Raman vibration peaks observed in the theoretical Raman spectrum of SM2, the Raman spectrum of the SM2 standard product, and the SERS spectrum of the SM2 aqueous solution show good consistency. The theoretical Raman vibration peaks of SM2 are at 442 cm^−1^, 560 cm^−1^, 578 cm^−1^, 648 cm^−1^, 836 cm^−1^, 1012 cm^−1^, 1300 cm^−1^, 1370 cm^−1^, and 1596 cm^−1^. The Raman vibration peaks of the SM2 standard product are at 439 cm^−1^, 563 cm^−1^, 580 cm^−1^, 638 cm^−1^, 827 cm^−1^, 999 cm^−1^, 1306 cm^−1^, 1387 cm^−1^, and 1593 cm^−1^. The SERS vibration peaks of the SM2 aqueous solution are at 442 cm^−1^, 556 cm^−1^, 588 cm^−1^, 634 cm^−1^, 828 cm^−1^, 999 cm^−1^, 1306 cm^−1^, 1387 cm^−1^, and 1593 cm^−1^. The deviations among these three sets of peaks range from a minimum of 0 cm^−1^ to a maximum of 17 cm^−1^. This was because the theoretical spectrum of SM2 was calculated in the ideal vibrational mode, in which only the interaction between the molecular functional groups was calculated, and the intermolecular force was ignored, resulting in a certain deviation between the measured spectrum and the calculated theoretical spectrum.

The molecular formula of SM2 is C_12_H_14_N_4_O_2_S. The optimized molecular structure of SM2 primarily comprises several functional groups, including O=S=O, C=N-C, C-H, -CH3, and N-H. These functional groups can generate various vibrational bands through stretching, swinging (or more commonly, bending), and deforming. The positions of certain characteristic peaks of SM2 have been assigned and are presented in Table 1. The vibrational peak at 442 cm^−1^ can be assigned to the in-plane rocking vibrations of N-H and C-H bonds. The peak observed at 560 cm^−1^ is attributed to the S=O stretching vibration coupled with in-plane rocking of C-C-C and out-of-plane rocking of C-H. At 578 cm^−1^, the vibrational mode primarily involves in-plane C-C-C rocking and C-H out-of-plane deformation. The 648 cm^−1^ feature corresponds to in-plane C-C rocking vibrations, while the 836 cm^−1^ peak arises from C-H curling vibration. The characteristic peak at 1012 cm^−1^ results from C-C-N scissoring vibrations. For the 1300 cm^−1^ band, it demonstrates combined contributions from S=O stretching and N-H in-plane rocking. The vibrational mode at 1370 cm^−1^ incorporates C-C stretching coordinated with C-H out-of-plane deformation. Finally, the prominent peak at 1596 cm^−1^ is characteristic of C-N stretching vibrations [30,31,32].

**Figure 2:**
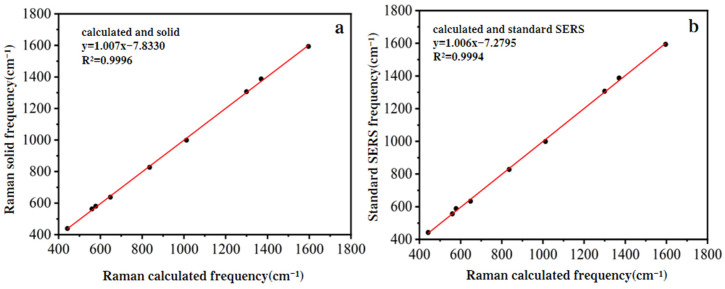
Linear fitting of theoretical and experimental Raman frequencies of SM2. (**a**) Solid standard and theoretical Raman frequency linear fit; (**b**) 5 mg/L standard solution SERS and theoretical Raman frequency linear fit.

In Figure 2, the theoretically calculated Raman shifts of the nine characteristic peaks listed in Table 1 are presented. The fitting curves compared these theoretically calculated Raman shifts with those of the standard solid and the SERS Raman shifts of the standard solution at a concentration of 5 mg/L. The fitting correlation coefficients (R^2^) were 0.9996 and 0.9994, respectively, indicating that the theoretically calculated spectrum provided reliable theoretical support for the experimental Raman spectrum of SM2.

### 3.2. SERS Signal Analysis of Duck Blood Samples Containing SM2 on Different Enhanced Substrates

Duck blood samples containing 5 mg/L SM2 and blank duck blood samples were mixed with laboratory-made 1 mM gold nanocolloids, 1% gold nanocolloids, 14th-generation gold nanocolloids, and silver nanocolloids, respectively. Their spectral data were then measured for comparison. Consistent detection parameters were ensured during the experimental process (the amount of enhanced substrate was 500 μL, the amount of sample to be tested was 20 μL, and the adsorption time was set to 1 min).

**Figure 3:**
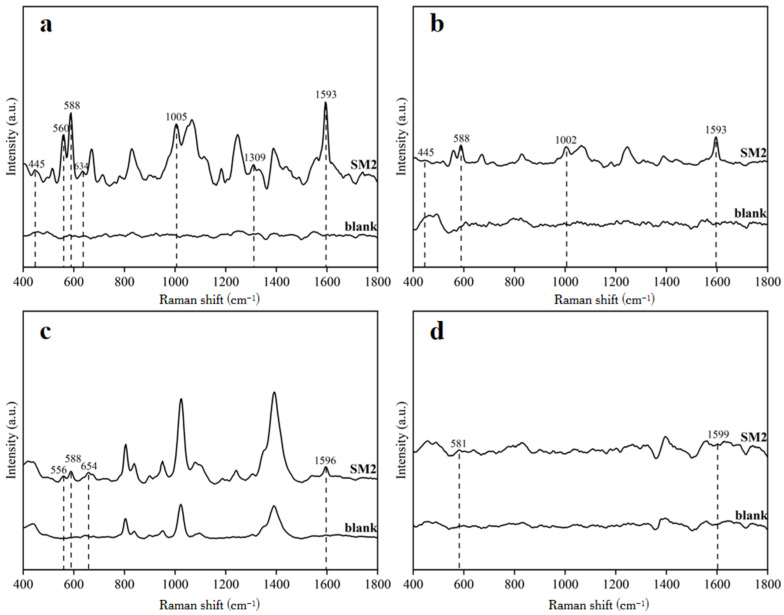
SERS spectra of duck blood samples containing SM2 on four enhanced bases: (**a**) 1 mM gold nanocolloids; (**b**) 1% gold nanocolloids; (**c**) 14th-generation gold nanocolloids; (**d**) silver nanocolloids.

As shown in Figure 3, the SM2 curve represents the SERS spectrum of the duck blood sample containing 5 mg/L SM2 using the corresponding enhanced substrate, while the blank curve represents the SERS spectrum of the duck blood sample without SM2. From the comparison diagram, it could be seen that duck blood samples containing SM2 exhibited four Raman characteristic peaks at 445 cm^−1^, 588 cm^−1^, 1002 cm^−1^, and 1593 cm^−1^ using a 1% concentration of gold nanocolloids. When 14th-generation gold nanocolloids were applied, the duck blood samples containing SM2 showed four characteristic Raman peaks at 556 cm^−1^, 588 cm^−1^, 654 cm^−1^, and 1596 cm^−1^, while three additional vibrational peaks were observed at 805 cm^−1^, 1021 cm^−1^, and 1393 cm^−1^. Significantly, the blank duck blood samples also displayed these three vibrational peaks at 805 cm^−1^, 1021 cm^−1^, and 1393 cm^−1^ under the same experimental conditions. These findings clearly indicated that these three peaks originated from the intrinsic background signals of the duck blood matrix itself rather than representing characteristic peaks of SM2. Therefore, these matrix-derived peaks could not serve as reliable indicators for determining the presence of SM2 in the test samples. When using silver nanocolloids, two Raman characteristic peaks were observed at 581 cm^−1^ and 1599 cm^−1^. In the case of 1 mM gold nanocolloids, seven Raman characteristic peaks were observed at 455 cm^−1^, 560 cm^−1^, 588 cm^−1^, 634 cm^−1^, 1005 cm^−1^, 1309 cm^−1^, and 1593 cm^−1^. The SERS spectra clearly showed that the 1 mM gold nanocolloid solution could excite a greater number and higher intensities of Raman characteristic peaks in duck blood samples containing SM2 compared to the blank samples.

### 3.3. Research on the Optimization of SERS Detection Conditions

#### 3.3.1. SERS Analysis of SM2 in Duck Blood

**Figure 4:**
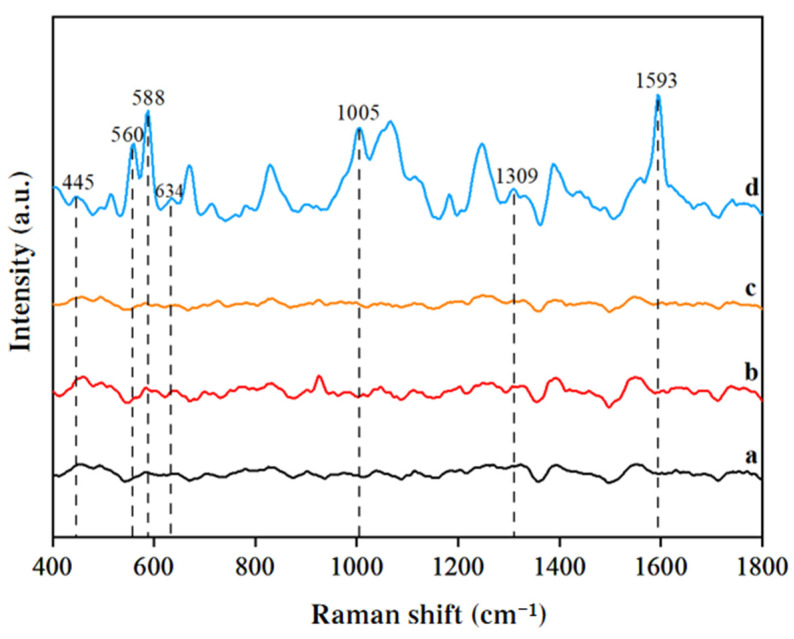
SERS spectrum comparison of duck blood samples containing 5 mg/L SM2: (**a**) 1 mM gold nanocolloids + ultrapure water + 0.1 mol/L CaCl_2_ solution; (**b**) 1 mM gold nanocolloids + 0.1% formic acid acetonitrile solution + 0.1 mol/L CaCl_2_ solution; (**c**) 1 mM gold nanocolloids + blank duck blood sample + 0.1 mol/L CaCl_2_ solution; (**d**) 1 mM gold nanocolloids+containing 5 mg/L SM2 duck blood sample + 0.1 mol/L CaCl_2_ solution.

As shown in Figure 4, the SERS spectra of the following solutions were compared: 1 mM gold nanocolloids + ultrapure water + 0.1 mol/L CaCl_2_ solution, 1 mM gold nanocolloids + 0.1% formic acid in acetonitrile solution + 0.1 mol/L CaCl_2_ solution, 1 mM gold nanocolloids + blank duck blood sample + 0.1 mol/L CaCl_2_ solution, and 1 mM gold nanocolloids + duck blood sample containing 5 mg/L SM2 + 0.1 mol/L CaCl_2_ solution. The duck blood samples containing SM2 exhibited distinct Raman characteristic peaks at 445 cm^−1^, 560 cm^−1^, 588 cm^−1^, 634 cm^−1^, 1005 cm^−1^, 1309 cm^−1^, and 1593 cm^−1^. Among these, the peaks at 588 cm^−1^, 1005 cm^−1^, and 1593 cm^−1^ demonstrated significantly higher intensities and were, therefore, selected as the SERS signature peaks for identifying SM2 residues in duck blood.

#### 3.3.2. The Effect of the Ratio of Duck Blood Sample to Extractant on Raman Intensity

**Figure 5:**
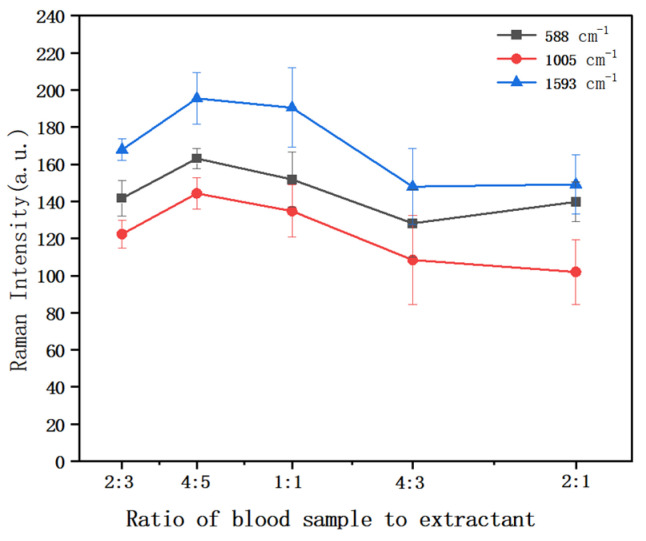
Comparison of characteristic peak intensity in different volume ratios of duck blood sample to extractant.

Duck blood is a red and opaque liquid. Its main components include water, plasma protein, inorganic salts, blood cells, and other cellular metabolites. Related studies have shown that the use of formic acid-acidified acetonitrile as an extractant can effectively improve the extraction efficiency of antibiotics and successfully capture the SERS signal of antibiotics. To eliminate the potential interference of proteins and other impurities on the SERS signal, a 0.1% formic acid acetonitrile solution was selected as the extractant in the pretreatment step. During the experiment, it was found that the ratio of duck blood sample to extractant had a significant effect on the final SERS signal intensity. Based on this finding, comparative experiments were carefully designed by selecting five different ratios (i.e., the volume ratios of duck blood samples to extractants were 2:3, 4:5, 1:1, 4:3, and 2:1, respectively), and duck blood samples containing 5 mg/L SM2 were prepared. The SERS spectra were analyzed in detail. By comparing and analyzing the data shown in Figure 5, it can be clearly seen that the duck blood samples containing 5 mg/L SM2 exhibited a consistent trend at the three characteristic peaks of 588 cm^−1^, 1005 cm^−1^, and 1593 cm^−1^. It is worth mentioning that the peak intensity of these characteristic peaks reached its maximum when the volume ratio of duck blood sample to extractant was 4:5. Therefore, in the subsequent experiments, the optimal ratio of duck blood sample to the extractant was determined to be 4:5.

#### 3.3.3. The Effects of Different Types of Agglomerates on Raman Intensity

**Figure 6:**
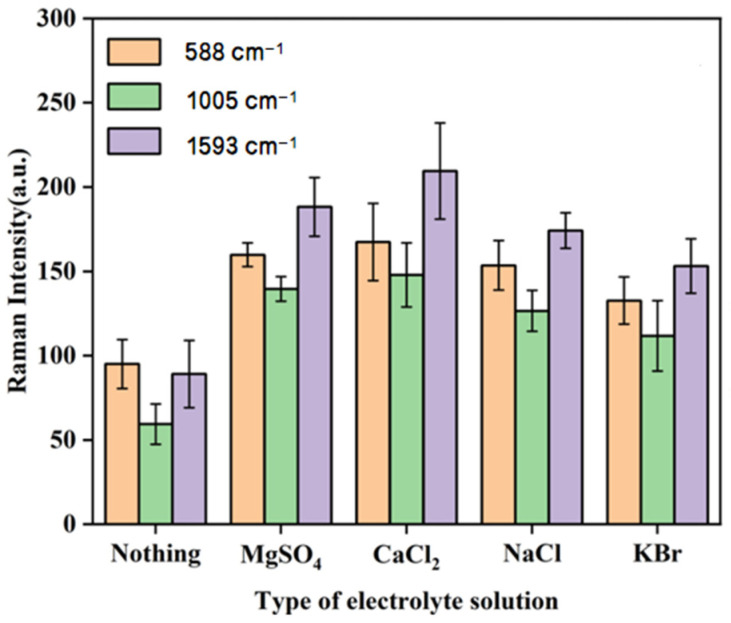
The effect of different electrolyte agglomerates on the SERS characteristic peaks of the samples.

Studies have shown that if electrolytes such as CaCl_2_, NaCl, and MgSO_4_ were selected as part of the SERS enhancement substrate, they could further enhance or reduce the Raman spectral intensity [33,34,35]. Therefore, the SERS enhancement effects of five enhanced substrate combinations (i.e., gold nanocolloids + ultrapure water, gold nanocolloids + MgSO_4_ solution, gold nanocolloids + CaCl_2_ solution, gold nanocolloids + NaCl solution, and gold nanocolloids + KBr solution) on samples containing SM2 were analyzed. Figure 6 shows that duck blood samples with 5 mg/L SM2 exhibited consistent trends at the 588 cm^−1^, 1005 cm^−1^, and 1593 cm^−1^ peaks. The peak intensity was maximized when CaCl_2_ solution was used as the electrolyte. Additionally, gold nanocolloids plus CaCl_2_ solution were more effective in stimulating SERS signals of SM2 in duck blood than gold nanocolloids alone. Based on the optimization results, the 1 mM gold nanocolloids combined with CaCl_2_ solution were ultimately selected as the enhanced substrate for surface-enhanced Raman spectroscopy (SERS) detection of SM2 residues in duck blood samples.

#### 3.3.4. The Effect of the Amount of Electrolyte Agglomeration Agent on Raman Intensity

The SERS activity of gold nanoparticles could be improved by adding electrolytes such as CaCl_2_, NaCl, and MgSO_4_ to the gold nanocolloids. Studies showed that the amount of electrolyte added also affected the SERS signal [36]. In the above experiments, we first determined that the best electrolyte was CaCl_2_. Subsequently, we investigated the effect of adding CaCl_2_ solution on the activity of gold nanocolloids.

**Figure 7:**
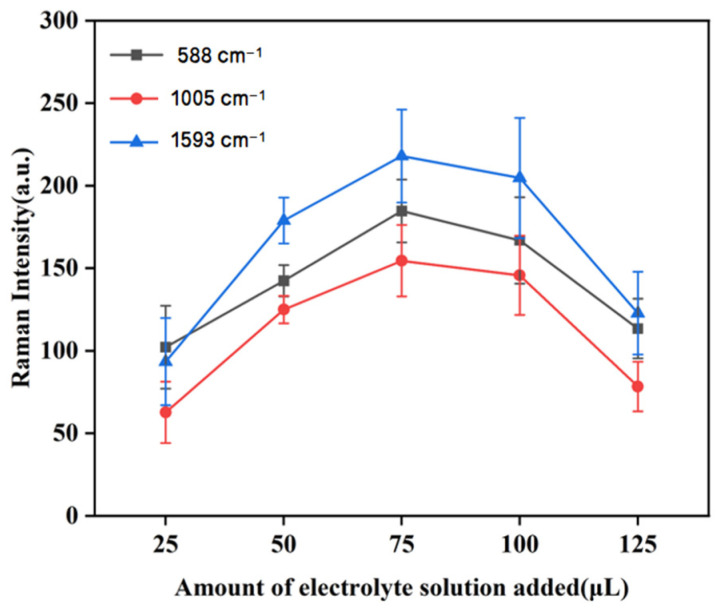
The effect of the amount of electrolyte agglomerators added on the SERS characteristic peak of samples.

As seen in Figure 7, the duck blood samples containing 5 mg/L of SM2 demonstrated a consistent trend at the three characteristic peaks of 588 cm^−1^, 1005 cm^−1^, and 1593 cm^−1^. The peak intensity reached its maximum when 75 μL of CaCl_2_ solution was added. From the trend diagram depicted in Figure 7, it was evident that the SERS signal of SM2 molecules progressively increased with the addition of a small amount of electrolyte. This enhancement could be attributed to the aggregation of nanoparticles, which facilitated an increase in SERS active sites. However, as the amount of electrolyte added continued to rise, the stable structure of the colloid was disrupted due to the excess electrolyte, resulting in the agglomeration of the colloid and subsequently causing the SERS signal to diminish or even vanish. Consequently, 75 μL of CaCl_2_ was determined to be the optimal amount of electrolyte to be added for the SERS detection of SM2 in duck blood samples.

#### 3.3.5. The Effect of Different Amounts of Gold Nanocolloids on Raman Intensity

**Figure 8:**
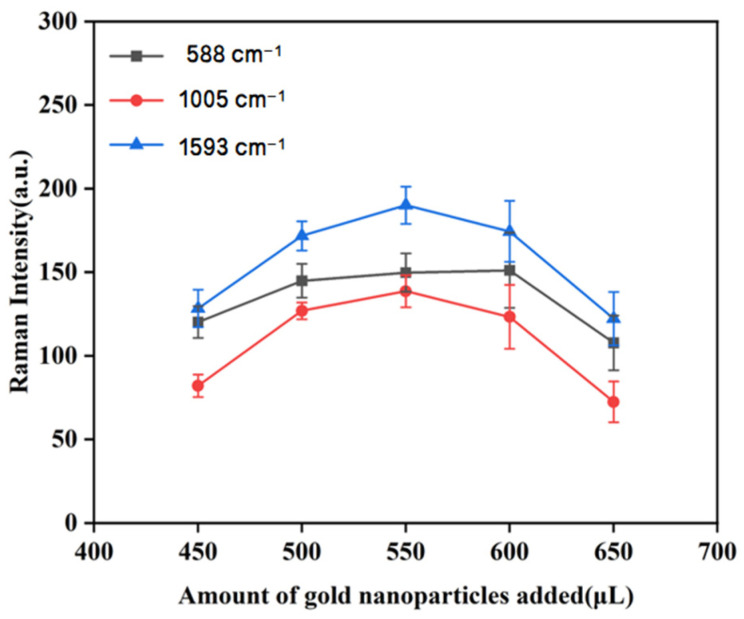
The effect of the amount of gold nanocolloids added on the SERS characteristic peak of samples.

When sample solution molecules encountered gold nanoparticles, a significant number of these molecules aggregated and adsorbed in the gaps of the rough surface of the gold nanoparticles, thereby enhancing the SERS spectrum. Past studies have shown that varying the amount of gold nanocolloids added could affect the SERS signal intensity. Therefore, it was necessary to optimize the quantity of gold nanocolloids [37,38]. It can be seen from Figure 8 that when 550 μL of 1 mM gold nanocolloids was added to the duck blood sample containing 5 mg/L SM2, the peak intensity reached its maximum at 1005 cm^−1^ and 1593 cm^−1^. The peak intensity at 588 cm^−1^ was second only to that observed when 600 μL of 1 mM gold nanocolloids was added. A comprehensive comparison revealed that 550 μL of 1 mM gold nanocolloids had the best SERS enhancement effect on duck blood samples containing 5 mg/L SM2.

It is observed in Figure 8 that as the amount of gold nanocolloids increased, the intensity of the main characteristic peaks in duck blood samples containing SM2 exhibited a consistent trend: the intensity of the Raman characteristic peaks initially increased and then decreased. The possible reason is that when the amount of gold nanocolloids increased to a certain level, more SM2 molecules were adsorbed onto the surface of gold nanoparticles, making the SERS signal at the characteristic peaks the strongest. Subsequently, due to the excessive volume of gold nanoparticles, the surface area available for binding with the measured molecules on the gold nanoparticles was reduced, which inhibited the binding of further molecules. Therefore, 550 μL was selected as the optimal amount of gold nanocolloids in the SERS detection test of duck blood samples containing SM2.

#### 3.3.6. The Effect of Different Adsorption Times on Raman Intensity

When the analyte was adsorbed onto the surface of gold nanoparticles, the adsorption time played a crucial role in enhancing the intensity of the SERS signal. At a certain point during the contact between the analyte and the enhanced substrate, an active ‘hot spot’ was generated, maximizing the SERS signal [39,40].

**Figure 9:**
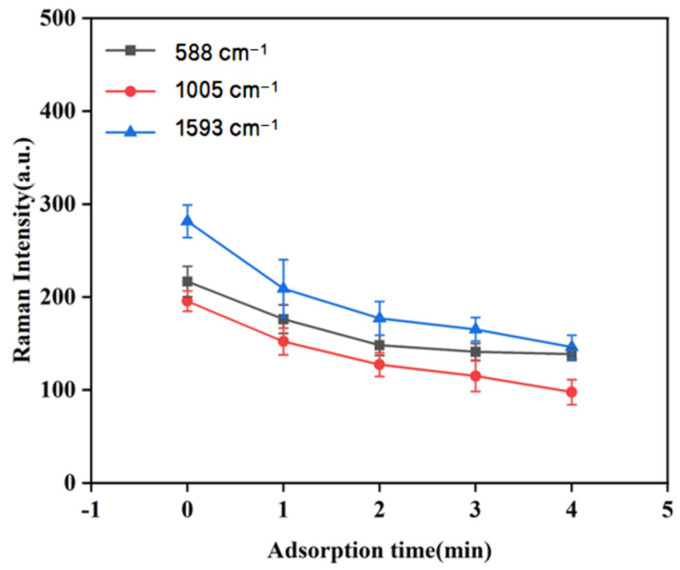
The effect of adsorption time on the SERS characteristic peaks of samples.

As can be seen from Figure 9, the duck blood samples containing 5 mg/L SM2 exhibited a consistent trend at the three characteristic peaks located at 588 cm^−1^, 1005 cm^−1^, and 1593 cm^−1^. When the adsorption time was 0 min, the peak intensity reached the maximum and then gradually decreased. In the early stage of contact, the SERS signal was the strongest, possibly due to the abundance of active hot spots on the surface. However, in the later stage of contact, the spectral intensity of SERS diminished as a result of the decreasing number of active hot spots. Additionally, operating at 0 min posed difficulties in practice, thereby resulting in significant errors. Therefore, in order to ensure the stability and accuracy of the test conditions, 1 min was selected as the best adsorption time for the antibiotic SERS detection test.

### 3.4. Quantitative Analysis of SM2 Residues in Duck Blood

#### 3.4.1. Raman Spectroscopy and Detection Limit Analysis of SM2 Residues in Duck Blood

**Figure 10:**
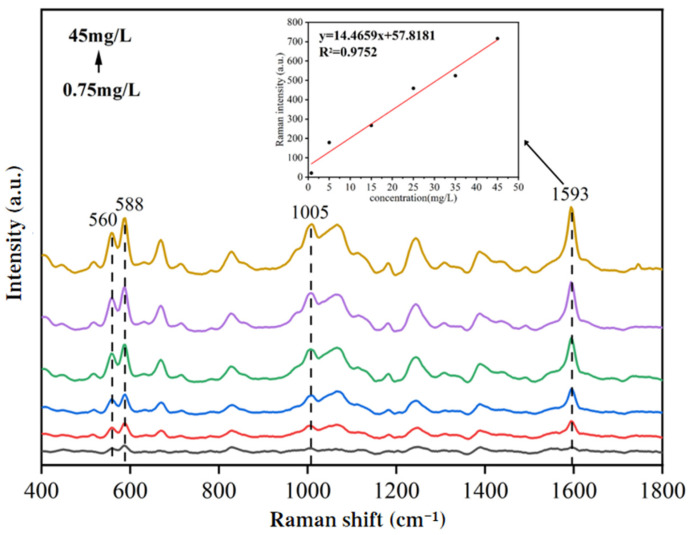
SERS spectra of duck blood samples containing different concentrations of SM2.

Figure 10 shows the SERS spectra of duck blood samples containing different concentrations (0.75 mg/L, 5 mg/L, 15 mg/L, 25 mg/L, 35 mg/L, 45 mg/L) of SM2. It can be seen from the figure that as the concentration of SM2 in duck blood increased, the intensities of characteristic peaks at Raman shifts such as 560 cm^−1^, 588 cm^−1^, 1005 cm^−1^, and 1593 cm^−1^ gradually increased. Using the peak intensity at 1593 cm^−1^ of duck blood samples containing SM2 as an example, the standard fitting curve of the characteristic peak intensity and concentration of SM2 in duck blood was established. The curve in Figure 10 was represented by the equation *y* = 14.4659*x* + 57.8181, with an R^2^ value of 0.9752. Based on the peak intensity at 1593 cm^−1^, SM2 residues in duck blood demonstrated a good linear relationship in the concentration range from 0.75 to 45.0 mg/L. When the concentration of SM2 was 0.75 mg/L, the intensity of the characteristic peak was very low, but it was still visible.

#### 3.4.2. Raman Spectroscopic Pretreatment of SM2 Residues in Duck Blood

In this study, seven pretreatment methods were employed: air-PLS, as well as air-PLS combined with first-order derivative, second-order derivative, normalization, SG, SNV, or MSC. These methods were used to preprocess the original spectral data, and subsequently, SVR, PLSR, and MLR models were established to analyze the Raman spectra of the samples. The optimal preprocessing method was determined based on the maximum R_p_^2^ and the minimum RMSEC in the established CARS-MLR model. As shown in Table 2, the models established using four of these spectral preprocessing methods—air-PLS, air-PLS combined with SG, air-PLS with the first derivative, and air-PLS with the second derivative—all exhibited an R_p_^2^ value above 0.96 and a small RMSEP value. After applying air-PLS pretreatment, the CARS-MLR prediction model achieved an R_p_^2^ of 0.9817 and an RMSEP of 1.5539, both of which were optimal. Therefore, air-PLS was selected as the spectral preprocessing method for the CARS-MLR prediction model of SM2 residues in duck blood.

#### 3.4.3. Extraction of Raman Spectral Features for SM2 Residues in Duck Blood

**Figure 11:**
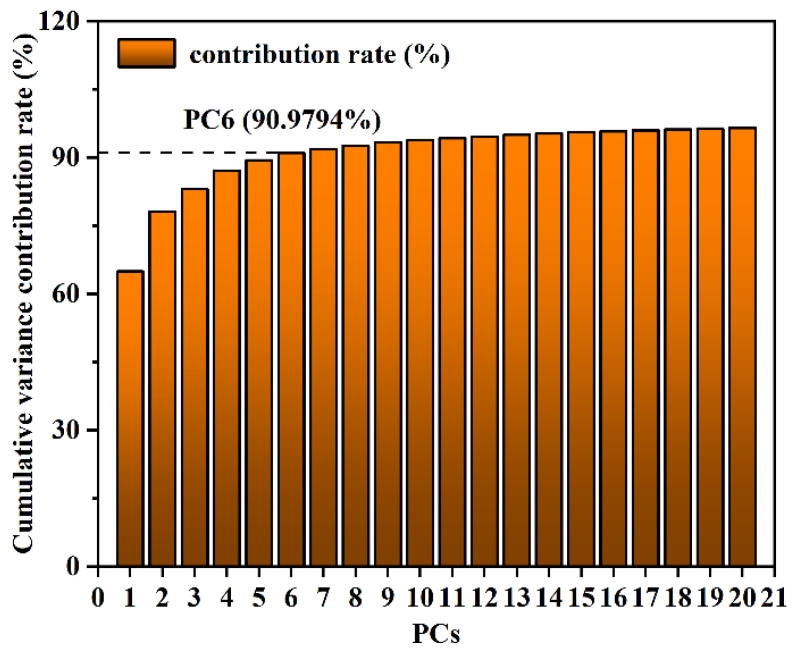
The effects of the number of principal component factors on the cumulative variance contribution rate.

**Figure 12:**
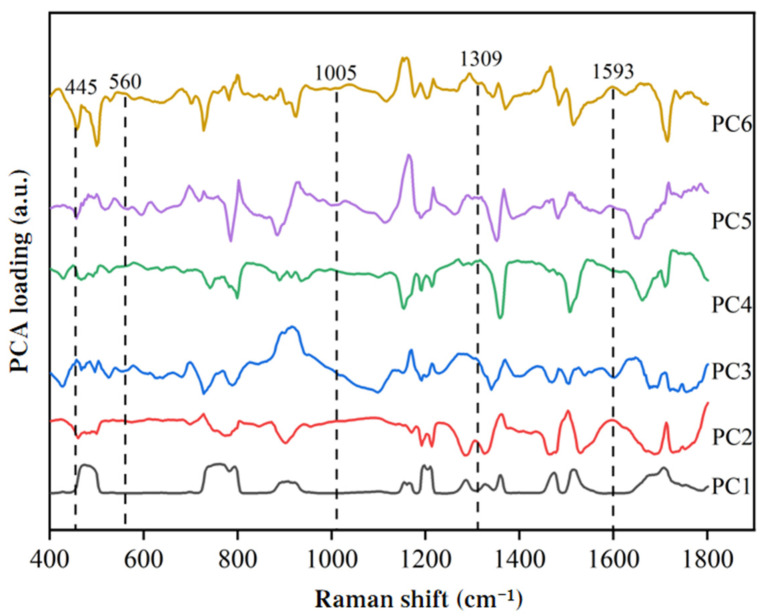
Loading distribution of the first 6 principal components of SM2 in duck blood based on PCA.

To reduce data redundancy and enhance modeling efficiency, this paper explored the use of two feature extraction methods, namely CARS and PCA, for extracting features from the SERS spectra of duck blood containing SM2. PCA feature extraction was applied to the initial 449 variables. As illustrated in Figure 11, the cumulative variance contribution rate of the first six principal components reached 90.9794%. Consequently, these six components were selected as the inputs for the MLR model. The loading data of the first six principal components were used to generate a PCA loading distribution plot, as shown in Figure 12. From the plot, it can be observed that PC3 and PC4 exhibited peaks at the characteristic peak of 445 cm^−1^, PC2 showed a peak at the characteristic peak of 1309 cm^−1^, and PC2 and PC6 displayed peaks at the characteristic peak of 1593 cm^−1^. This indicates that the spectral characteristic frequencies extracted through PCA could serve as input values for the MLR model.

**Figure 13:**
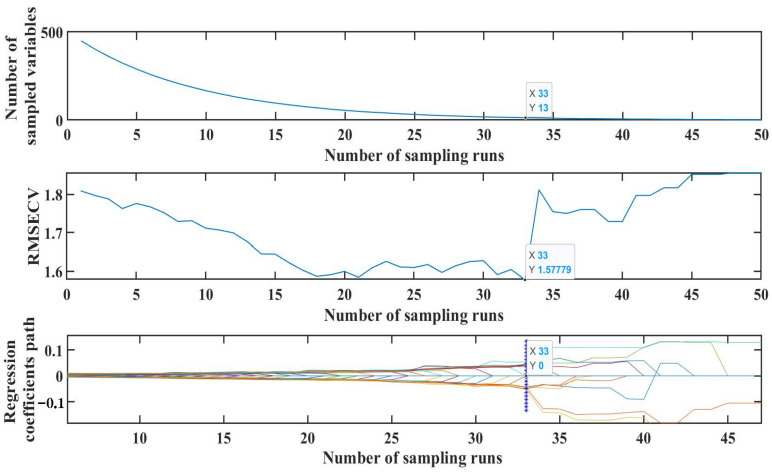
Characteristic extraction of SM2 from duck blood based on CARS.

When CARS was applied to extract features from the SERS spectra of duck blood containing SM2, the screening process and its details were illustrated in Figure 13. As the number of iterations accumulated, the count of variables in each iteration decreased. It was evident that the screening method was effective in selecting an appropriate number of variables from the extensive dataset. Further examination of the graphical data revealed that the root mean square error of the cross-validation (RMSECV) reached its lowest point when the number of iterations hit 33. At this juncture, a total of 13 characteristic bands were identified through screening, constituting approximately 2.8% of the total number of bands. Consequently, these 13 characteristic variables were successfully extracted using the CARS method and utilized as input values for the MLR model to predict SM2 residues in duck blood.

In Table 3, a comparison is presented of the performance of the MLR models established under two different conditions. After feature variables were extracted using CARS and PCA, the performance of the models was significantly improved. PCA extracted 6 feature variables from 449 variables as input values for the MLR model, achieving an R_p_^2^ of 0.9652. CARS extracted 13 feature variables from 449 variables as input values for the MLR model, achieving an R_p_^2^ of 0.9817, and the RMSEP was also better than that of PCA. Therefore, considering all factors, CARS was selected as the method for extracting feature variables in the quantitative prediction model of SM2 residues in duck blood.

#### 3.4.4. Quantitative Prediction Model of SM2 Residues in Duck Blood

**Figure 14:**
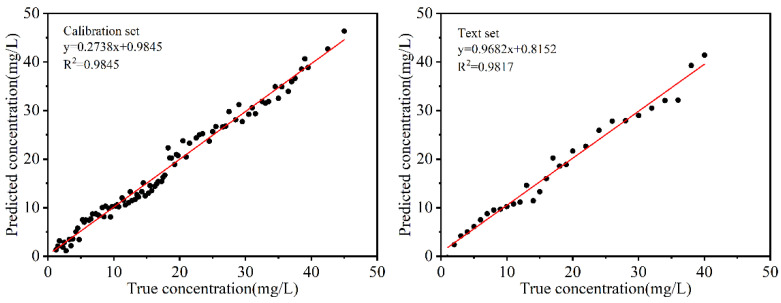
Linear fitting of true and predicted values.

After spectral preprocessing and feature extraction of the data, three modeling methods (SVR, MLR, and PLSR) were employed for analysis and comparison, with the aim of assessing the prediction accuracy and overall performance of the models. As shown in Table 4, the R_p_^2^, RMSEP, and RPD of the model based on MLR were 0.9817, 1.5539, and 7.1953, respectively, indicating that the MLR model performed the best among SVR, MLR, and PLSR. Figure 14 illustrates the prediction performance of both the training set and the prediction set for the established MLR model. The results indicate that the MLR model exhibits good predictability. In conclusion, the MLR-based prediction of SM2 residues in duck blood aligns closely with actual measurements, further confirming the model’s high prediction accuracy.

## 4. Conclusions

This study proposed a method for the detection of SM2 residues in duck blood. This method combined density functional theory to optimize the molecular structure of SM2 and performed theoretical Raman vibration analysis. Additionally, a comparative analysis was conducted to assess the enhancement effects of four different substrates: 1 mM gold nanocolloids, 1% gold nanocolloids, 14th-generation gold nanocolloids, and silver nanocolloids. The results indicate that the SERS enhancement effect of 1 mM gold nanocolloids on SM2 in duck blood was superior to that of the other three substrates. To meet the detection requirements for SM2 in duck blood, this study also thoroughly investigated and optimized several key parameters, including the sample pretreatment method, the type and amount of electrolyte agglomeration agent added, the amount of gold nanocolloids added, and the adsorption time. The optimal sample pretreatment method was as follows: the duck blood sample was mixed with 0.1% formic acid acetonitrile solution at a volume ratio of 4:5. For duck blood samples containing SM2, the optimum experimental conditions were determined as follows: CaCl_2_ was selected as the electrolyte aggregation agent; the addition volume was 75 μL; the volume of gold nanocolloid solution was 550 μL; and the adsorption time was 1 min. In the process of data processing, after comparing seven different spectral data preprocessing methods, air-PLS was finally selected as the optimal preprocessing method. With the help of a portable Raman spectrometer, this study successfully achieved the quantitative detection of SM2 residues in duck blood. Combined with chemometrics, a CARS-MLR model with excellent performance was established. The R_p_^2^ was 0.9817, RMSEC was 1.5539 mg/L, RPD was 7.1953, and the quantitative detection limit was as low as 0.75 mg/L. This method avoids the destruction of tissues that can be used in meat ducks, and it offers simple, low-cost, and high-efficiency sample collection. This provides a new direction for the rapid detection of antibiotic residues in meat ducks.

## Figures and Tables

**Table 1:** Attribution analysis of SM2 peak position.

Theoretical Raman (cm^−1^)	Solid Standard Raman Spectrum (cm^−1^)	SERS Spectra of Standard Aqueous Solution (cm^−1^)	Peak Position Attribution Analysis of SM2
442	439	442	N-H, C-H in-plane rocking vibration
560	563	556	S=O stretching vibration, C-C-C in-plane rocking vibration, C-H out-of-plane rocking vibration
578	580	588	C-C-C in-plane rocking vibration, C-H out-of-plane rocking vibration
648	638	634	C-C in-plane rocking vibration
836	827	828	C-H curling vibration
1012	999	999	C-C-N scissoring vibration
1300	1306	1306	S=O stretching vibration, N-H in-plane rocking vibration
1370	1387	1387	C-C stretching vibration, C-H out-of-plane rocking vibration
1596	1593	1593	C-N stretching vibration

**Table 2:** Results of CARS-MLR model under different spectral pretreatment methods.

Pretreatment Methods	Characteristic Number	Total Number of Variables	R_c_^2^	RMSEC	R_p_^2^	RMSEP
air-PLS	13	499	0.9845	1.5561	0.9817	1.5539
air-PLS + SG	7	499	0.9832	1.5601	0.9804	1.5982
air-PLS + first derivative	4	499	0.9767	1.8011	0.9679	2.0163
air-PLS + second derivative	9	499	0.9792	1.7565	0.9720	1.8830
air-PLS + normalization	40	499	0.9716	2.6199	2.6199	5.1382
air-PLS + SNV	32	499	0.9620	2.8114	2.8114	4.2437
air-PLS + MSC	44	499	0.9814	2.2145	2.2145	4.3097

**Table 3:** MLR model results of different feature extraction methods.

Feature Extraction Method	Feature Number	R_c_^2^	RMSEC	R_p_^2^	RMSEP
CARS + MLR	13	0.9845	1.5561	0.9817	1.5539
PCA + MLR	6	0.9719	2.0011	0.9652	2.1255

**Table 4:** Prediction performance analysis of different models.

Model	R_c_^2^	RMSEC	R_p_^2^	RMSEP	RPD
PLSR	0.9670	2.0855	0.9572	2.3369	4.7532
MLR	0.9845	1.5561	0.9817	1.5539	7.1953
SVR	0.9797	1.6354	0.9724	1.9225	5.6295

## Data Availability

The original contributions presented in this study are included in the article. Further inquiries can be directed to the corresponding author.
